# Echinochrome Ameliorates Physiological, Immunological, and Histopathological Alterations Induced by Ovalbumin in Asthmatic Mice by Modulating the Keap1/Nrf2 Signaling Pathway

**DOI:** 10.3390/md21080455

**Published:** 2023-08-18

**Authors:** Islam Ahmed Abdelmawgood, Noha Ahmed Mahana, Abeer Mahmoud Badr, Ayman Saber Mohamed, Abdeljalil Mohamed Al Shawoush, Tarek Atia, Amir Elhadi Abdelrazak, Hader I. Sakr

**Affiliations:** 1 Zoology Department, Faculty of Science, Cairo University, Giza 12613, Egypt; 2 Zoology Department, Faculty of Science, Sirte University, Sirte 128123, Libya; sedra200713@yahoo.com; 3 Department of Medical Laboratory Sciences, College of Applied Medical Sciences, Prince Sattam bin Abdulaziz University, Al-Kharj 11942, Saudi Arabia; t.mohamed@psau.edu.sa; 4 Department of Medical Physiology, Medicine Program, Batterjee Medical College, Jeddah 21442, Saudi Arabia; physiology10.jed@bmc.edu.sa (A.E.A.); hadersakr@kasralainy.edu.eg (H.I.S.); 5 Department of Medical Physiology, Faculty of Medicine, Cairo University, Cairo 11562, Egypt

**Keywords:** echinochrome, asthma, ovalbumin, oxidative stress, inflammation, Keap1, Nrf2

## Abstract

Asthma is a persistent inflammatory disease of the bronchi characterized by oxidative stress, airway remodeling, and inflammation. Echinochrome (Ech) is a dark-red pigment with antioxidant and anti-inflammatory activities. In this research, we aimed to investigate the effects of Ech against asthma-induced inflammation, oxidative stress, and histopathological alterations in the spleen, liver, and kidney in mice. Mice were divided into four groups (*n* = 8 for each): control, asthmatic, and asthmatic mice treated intraperitoneally with 0.1 and 1 mg/kg of Ech. In vitro, findings confirmed the antioxidant and anti-inflammatory activities of Ech. Ech showed antiasthmatic effects by lowering the serum levels of immunoglobulin E (IgE), interleukin 4 (IL-4), and interleukin 1β (IL-1β). It attenuated oxidative stress by lowering malondialdehyde (MDA) and nitric oxide (NO) contents and increasing reduced glutathione (GSH), superoxide dismutase (SOD), glutathione-s-transferase (GST), and catalase (CAT) in the liver, spleen, and kidney. Moreover, it protected asthma-induced kidney and liver functions by increasing total protein and albumin and decreasing aspartate aminotransferase (AST), alanine aminotransferase (ALT), creatinine, urea, and uric acid levels. Additionally, it ameliorated histopathological abnormalities in the lung, liver, spleen, and kidney. Additionally, molecular docking studies were used to examine the interactions between Ech and Kelch-like ECH-associated protein 1 (Keap1). PCR and Western blot analyses confirmed the association of Ech with Keap1 and, consequently, the regulatory role of Ech in the Keap1-(nuclear factor erythroid 2-related factor 2) Nrf2 signaling pathway in the liver, spleen, and kidney. According to our findings, Ech prevented asthma and its complications in the spleen, liver, and kidney. Inhibition of inflammation and oxidative stress are two of echinochrome’s therapeutic actions in managing asthma by modulating the Keap1/Nrf2 signaling pathway.

## 1. Introduction

Asthma is a common chronic lung condition characterized by bronchial hyperreactivity, inflammation, and structural and functional alterations of the airways. Asthma affects approximately 235 million individuals globally [1]. Asthma development has been reported to be significantly influenced by differences in Th1/Th2 responses. Increased production of Th2-type cytokines, such as interleukin (IL)-4, IL-5, and IL-13, has been associated with uncontrolled Th2 immune responses. These cytokines cause tissue structure and function changes, eosinophil infiltration, bronchial inflammation, mucus hyperproduction, and immunoglobulin E (IgE) synthesis [2,3]. IL-4 drives the polarization of T lymphocytes towards Th2 cells, which in turn triggers the generation of more IL-4, which stimulates the switch of B cells to IgE release and the infiltration of mast cells and eosinophils [4]. Combining these effects results in airway constriction and hyperresponsiveness. Therefore, reducing the levels of these cytokines may alleviate asthmatic symptoms. 

Additionally, the pathogenesis of asthma is greatly affected by oxidative stress, and the asthmatic condition is frequently associated with oxidative stress [5]. Oxidative stress harms liver and kidney function [6,7]. Oxidative stress, an essential contributor to liver disorders, develops when there are elevated levels of reactive oxygen species (ROS), and this will finally result in the loss of homeostasis [8]. The pathophysiology of kidney diseases has been associated with excessive ROS production and depletion of antioxidant defense mechanisms, resulting in tissue injury through various processes, including the propagation of lipid peroxidation [9]. The spleen is an essential supporting organ for maintaining bodily homeostasis. The spleen, as an essential part of the body’s immune system, is a barrier and blood filter [10]. Although it is the most fundamental organ to experience stress, there are no longer any statistics on it when oxidative stress is present. Therefore, it is critical to attenuate oxidative stress and its deleterious impact on the body’s organs.

One of the most crucial defensive systems against oxidative stress intimately linked to inflammatory illnesses is the Kelch-like ECH-associated protein 1/nuclear factor erythroid 2-related factor 2 (Keap1/Nrf2) pathway [11]. By controlling Keap1, Nrf2 exerts potent anti-inflammatory effects. Since many diseases are characterized by oxidative stress and inflammation, pharmacological stimulation of Nrf2 is a potentially effective therapeutic method for treating and preventing many conditions [12]. Echinochrome (Ech)’s possible binding mechanism with Keap1 is to disrupt the Keap1/Nrf2 protein–protein interaction and activate Nrf2 signaling, which was investigated using molecular docking simulations.

To manage symptoms, enhance pulmonary function, and improve patient outcomes, inhaled corticosteroids (ICS) and bronchodilators are the recommended primary medications for respiratory problems. Nevertheless, prolonged ICS administration has been linked to evident harmful effects [13]. Surprisingly, most marine species have various pharmacological actions, including antibacterial, antiviral, antiparasitic, anti-inflammatory, and antidiabetic properties [14]. Ech, a naturally occurring pigment extracted from the shells and spines of sea urchins, has been shown to have antioxidant properties and to be helpful in various diseases [15]. A previous study showed that Ech possesses antifibrotic and anti-inflammatory properties by suppressing fibroblast stimulation and proinflammatory cytokine expression [16]. It has been reported that Ech treatment showed protective effects on the kidney and liver by eliminating the production of ROS in these tissues [17,18]. Therefore, the purpose of the current research was to determine the antiasthmatic mechanisms of Ech on the liver, kidney, and spleen of asthmatic mice.

## 2. Results

### 2.1. High-Performance Liquid Chromatography (HPLC)

**Figure 1:**
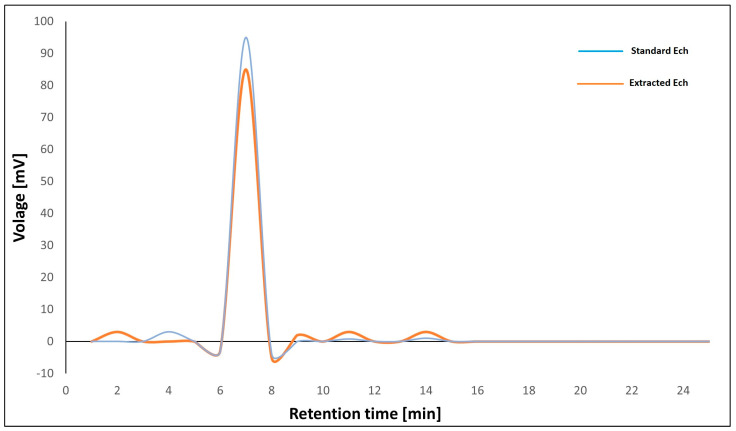
HPLC chromatograph of standard Ech and extracted Ech from sea urchin.

As illustrated in Figure 1, the HPLC analyses of separated Ech showed a notable peak with a retention period of 7.11 min, which is the same as the standard Ech with a total concentration of 85.5%.

### 2.2. In Vitro Antioxidant and Anti-Inflammatory Activities

**Figure 2:**
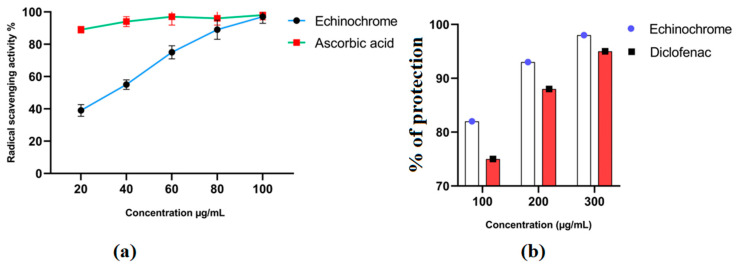
In vitro biological activities of Ech. (**a**) Antioxidant activity measured using 2,2-Diphenyl-1-picrylhydrazyl (DPPH) assay. (**b**) Anti-inflammatory activity using heat-induced hemolysis.

In vitro studies confirmed the antioxidant and anti-inflammatory properties of Ech. Ech’s antioxidant properties were dose-dependent (Figure 2a). The anti-inflammatory activity of Ech was validated by its ability to protect the membrane of red blood cells from heat-induced hemolysis at a range of concentrations (Figure 2b).

### 2.3. Molecular Docking Interaction between Ech and Keap1

**Figure 3:**
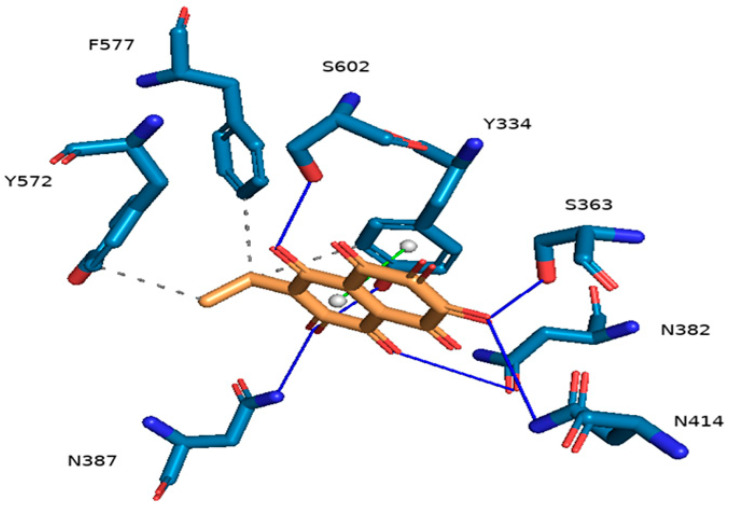
Three-dimensional visualization of Keap1–Ech binding complex showcasing favorable steric interactions, including a total of six hydrogen bonds with target residues Y334, S363, N382, N387, N414, and S602, strong hydrophobic interactions with Y334, Y572, and F577, and π–π stacking interaction with the aromatic ring of Y334.

**Figure 4:**
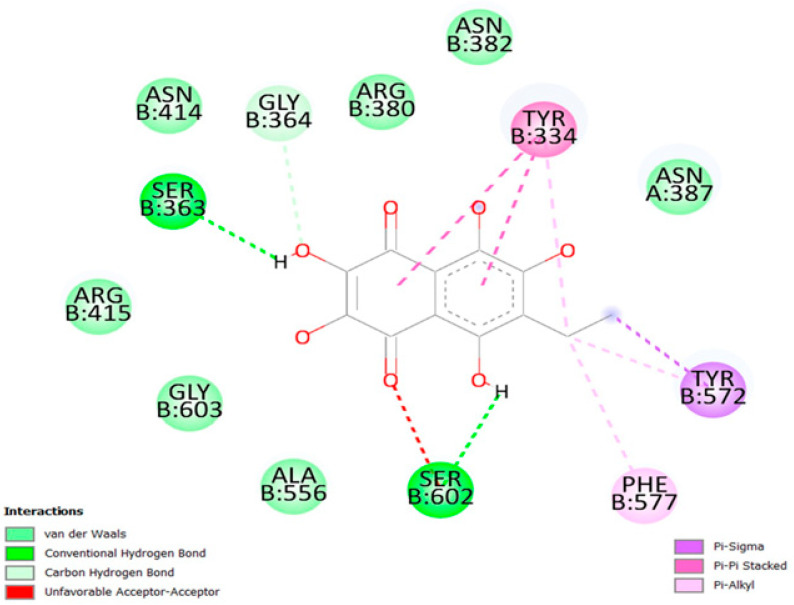
Two-dimensional visualization of the Keap1–Ech complex showcasing a total of two hydrogen bonds with binding site residues S363 and S602 as well as a total of seven hydrophobic interactions with residues N382, N387, N414, R415, A556, and G603, and pi-stacking with the aromatic ring of Y334.

Grid box dimensions in Table 1 were used in the docking of Ech to Keap1. The docking score of Ech binding with Keap1 was −7.9 kcal/mol. Ech three-dimensional visualization showcased several favorable interactions in the binding site of the target protein, including several hydrogen bonds, hydrophobic interactions, and π-stacking. Visualization of the protein–ligand complex revealed a total of six hydrogen bonds with target residues Y334, S363, N382, N387, N414, and S602; it exhibited strong hydrophobic interactions with Y334, Y572, and F577. Ech’s aromatic ring forms π–π stacking with the aromatic ring of Y334 (Figure 3). Two-dimensional visualization of the protein–ligand complex confirmed the three-dimensional results (Figure 4). Ech formed two hydrogen bonds with residues S363 and S602 in addition to seven hydrophobic interactions with residues R380, N382, N387, N414, R415, A556, and G603. Pi-stacking was also present between the aromatic rings of Ech and Y334.

### 2.4. Effect of Ech on Morphology, Body Weight, and Nasal Scratching

**Figure 5:**
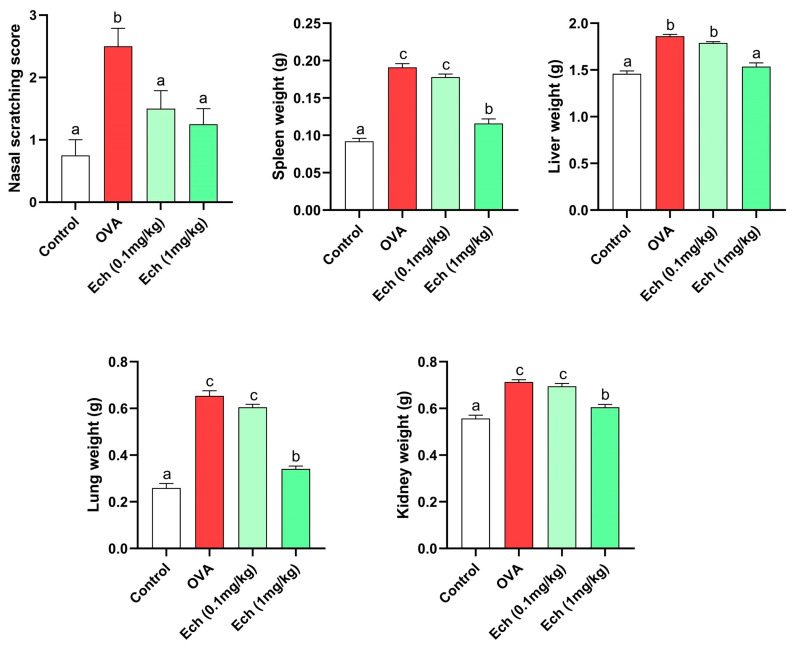
The effect of Ech on nasal scratching, lung, liver, kidney, and spleen weights. Values are given as means for 8 mice in each group ± standard error of the mean (SEM). The value that does not share a common letter superscript is significantly different (*p* < 0.05).

**Figure 6:**
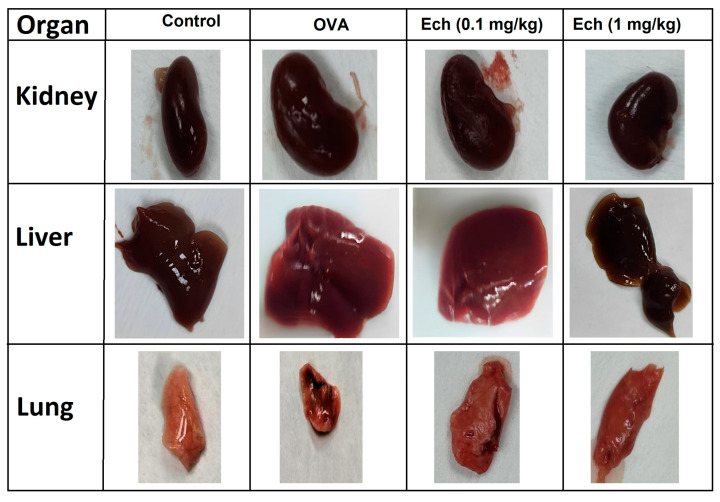
Organ morphology of the different groups.

The lung, liver, spleen, and kidney weights in OVA-challenged animals increased significantly compared to control animals. Remarkably, treatment with Ech (1 mg/kg) significantly (*p* > 0.05) improved the weight of the organs (Figure 5). Moreover, the OVA group had a significantly (*p* < 0.05) higher nasal scratching score than the control groups. Compared to the OVA group, the nasal scratching score significantly decreased after treatment with the high dose of Ech. The overall organ morphologies are represented in Figure 6.

### 2.5. Ech Reduced the Serum Levels of IgE, IL-4, and IL-1β

**Figure 7:**
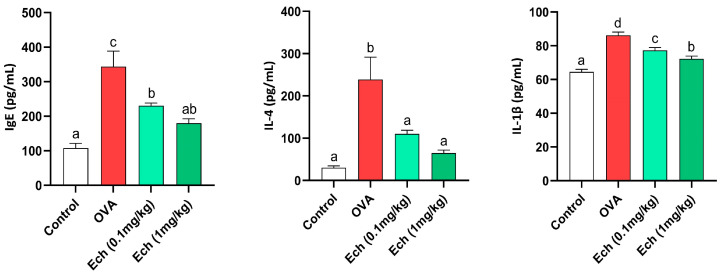
The effect of Ech on the serum levels of IgE, IL-4, and IL-1β. Values are given as means for 8 mice in each group ± standard error of the mean (SEM). The value that does not share a common letter superscript is significantly different (*p* < 0.05).

IgE, IL-4, and IL-1β levels increased significantly (*p* < 0.05) in OVA mice. However, Ech treatment dose-dependently reduced their concentrations, as shown in Figure 7.

### 2.6. Effect of Ech on Liver and Kidney Function

**Figure 8:**
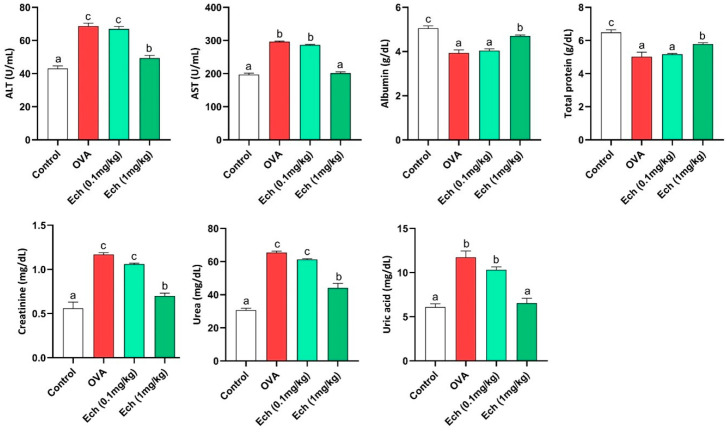
The effect of Ech on the liver functions parameters (AST, ALT, total protein, and albumin) and kidney function parameters (creatinine, urea, and uric acid). Values are given as means for 8 mice in each group ± standard error of the mean (SEM). The value that does not share a common letter superscript is significantly different (*p* < 0.05).

As shown in Figure 8, the OVA-challenged mice showed significantly higher levels of aspartate aminotransferase (AST), alanine aminotransferase (ALT), creatinine, uric acid, and urea compared to the control animals, with significantly lower levels of albumin and total protein. No noticeable difference was observed after treatment with 0.1 mg/kg Ech. In contrast to the OVA group, 1 mg/kg of Ech treatment significantly improved the liver and kidney function biomarkers (*p* < 0.05).

### 2.7. Effect of Ech on Keap1 and Nrf2 Protein Levels in the Liver, Kidney, and Spleen of Asthmatic Mice

**Figure 9:**
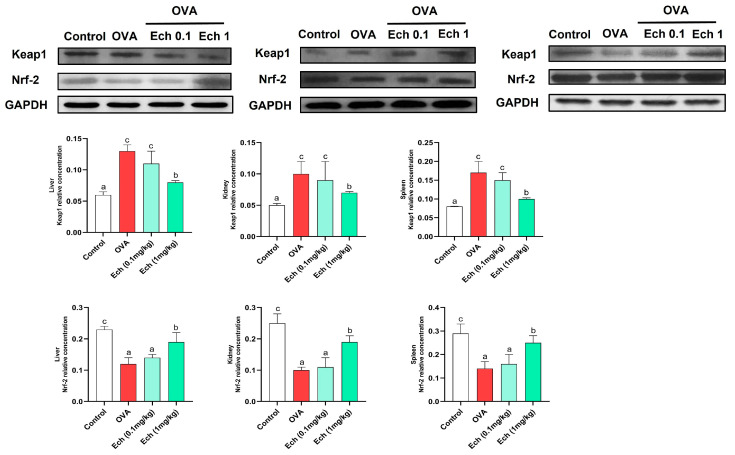
The effect of Ech on Keap1 and Nrf2 expression in the liver, kidney, and spleen of mice. The levels of Keap1 and Nrf2 proteins in the liver, kidney, and spleen were evaluated using Western blot with anti-GAPDH as a loading control. Values are given as means for 8 mice in each group ± standard error of the mean (SEM). The value that does not share a common letter superscript is significantly different (*p* < 0.05).

To further evaluate the therapeutic effect of Ech, we measured the expression of Keap1 and Nrf2 in the liver, kidney, and spleen (Figure 9). In the OVA group, Keap1 expression was considerably upregulated (*p* < 0.05), but Nrf2 expression was significantly downregulated (*p* < 0.05). Keap1 protein levels were significantly decreased, and Nrf2 levels were significantly increased after administering Ech (high dose). These findings suggested that Ech could regulate the Keap1/Nrf2 signaling pathway with anti-inflammatory and antioxidant properties.

### 2.8. Effects of Ech on the Expression of Keap1 and Nrf2 Genes in the Liver, Kidney, and Spleen of Asthmatic Mice

**Figure 10:**
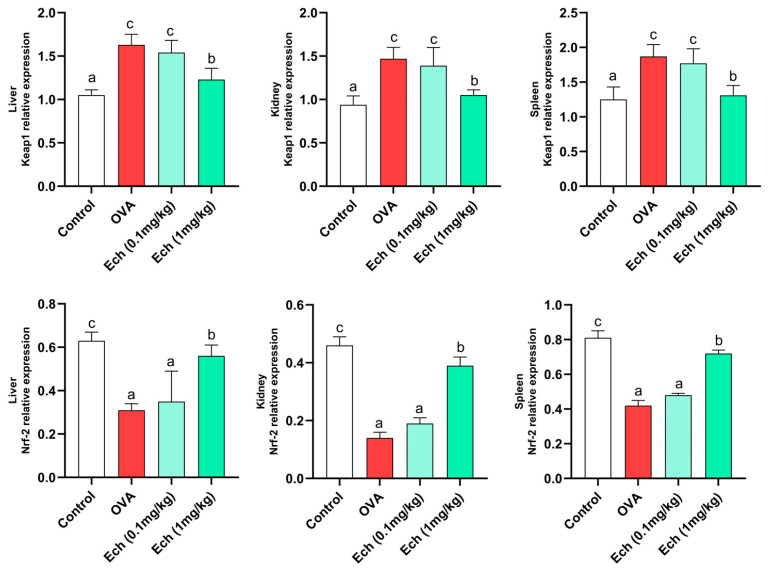
The effect of Ech on Keap1/Nrf2 pathway expression. The Keap1 and Nrf2 mRNA levels in the liver, kidney, and spleen were evaluated using RT-PCR with β-actin as an internal standard. Values are given as means for 8 mice in each group ± standard error of the mean (SEM). The value that does not share a common letter superscript is significantly different (*p* < 0.05).

According to real-time PCR results, the Nrf2 mRNA level in the OVA-challenged group was significantly (*p* < 0.05) lower than in the control group, whereas the Keap1 mRNA level was increased. Keap1 expression was suppressed, whereas Nrf2 expression was greatly boosted by the high dose of Ech (Figure 10).

### 2.9. Effect of Ech on Kidney Antioxidants and Oxidative Stress Markers

**Figure 11:**
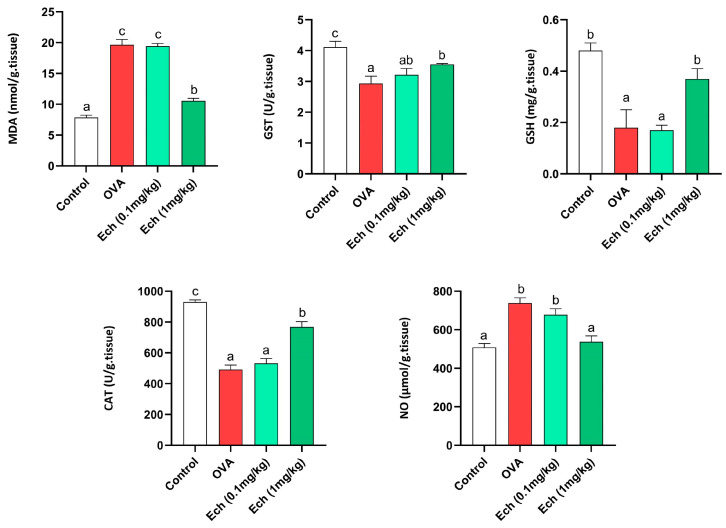
The effect of Ech on the kidney MDA, NO, and GSH levels and GST and CAT activity levels. Values are given as means for 8 mice in each group ± standard error of the mean (SEM). The value that does not share a common letter superscript is significantly different (*p* < 0.05).

Data concerning kidney antioxidants and oxidative stress markers such as MDA, NO, GSH, GST, and CAT are illustrated in Figure 11. The amounts of MDA, GSH, and NO were elevated in the OVA group. Also, the activity levels of GST and CAT were reduced in the OVA mice. No significant change was observed after treatment with 0.1 mg/kg Ech compared to the asthmatic mice. However, 1 mg/kg Ech treatment significantly decreased their levels (*p* < 0.05) compared to the OVA group.

### 2.10. Effect of Ech on Liver Antioxidants and Oxidative Stress Markers

**Figure 12:**
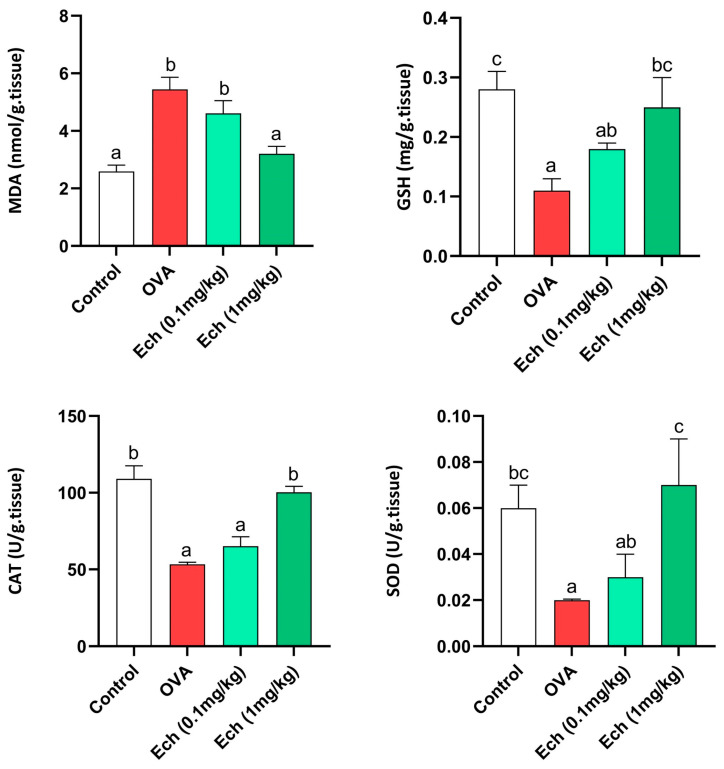
The effect of Ech on the liver’s MDA and GSH levels and SOD and CAT activity levels. Values are given as means for 8 mice in each group ± standard error of the mean (SEM). The value that does not share a common letter superscript is significantly different (*p* < 0.05).

Data concerning liver antioxidants and oxidative stress markers such as MDA, GSH, CAT, and SOD are shown in Figure 12. MDA and GSH were elevated in the OVA group compared to the control. Furthermore, the activity of CAT and SOD was reduced in the OVA group. Treatment with 0.1 mg/kg Ech showed no significant change in the OVA group. However, 1 mg/kg Ech treatment significantly decreased their concentrations compared to the OVA-challenged group (*p* < 0.05).

### 2.11. Effect of Ech on Spleen Antioxidants and Oxidative Stress Markers

**Figure 13:**
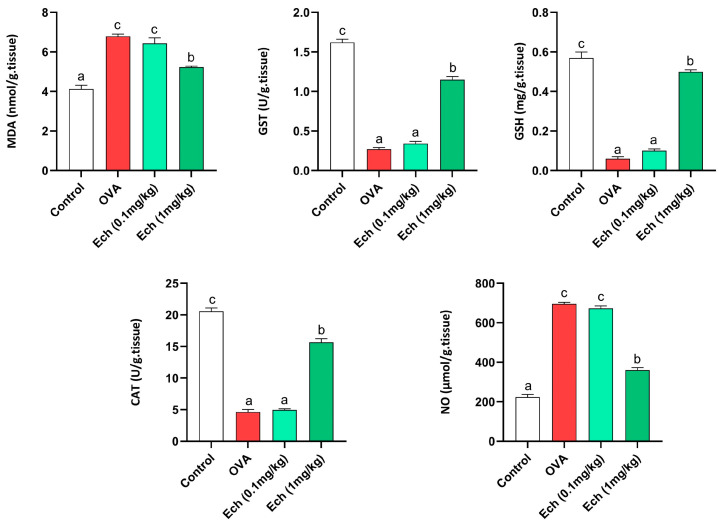
The effect of Ech on the spleen’s MDA, NO, and GSH levels and GST and CAT activity levels. Values are given as means for 8 mice in each group ± standard error of the mean (SEM). The value that does not share a common letter superscript is significantly different (*p* < 0.05).

Data concerning spleen antioxidants and oxidative stress markers (MDA, NO, GSH, GST, and CAT) are shown in Figure 13. The MDA, GSH, and NO levels were elevated in the OVA group. Moreover, the activity levels of GST and CAT were diminished in the OVA group. Intraperitoneal injection of 0.1 mg/kg Ech showed no significant change in the OVA-challenged group. However, 1 mg/kg Ech treatment notably diminished their levels (*p* < 0.05) compared to the OVA-challenged mice.

### 2.12. Effect of Ech on Lung Histopathology

**Figure 14:**
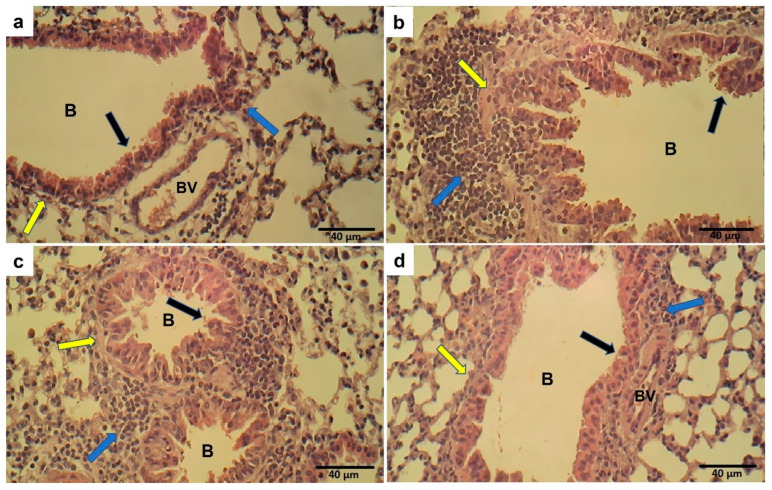
Lung sections of the control group (**a**) showed normal bronchi (B) with average epithelial lining (black arrow), average airway smooth muscles (yellow arrow), and minimal infiltrating immune cells (blue arrow). Sections from a mouse in the OVA group showed thickness in the bronchial epithelium (black arrow) and smooth muscle (yellow arrow) with marked infiltrating inflammatory cells (blue arrow) (**b**). Moderate cell infiltration, bronchial epithelium, and smooth muscle thickness were noticed in the 0.1 mg/kg Ech-treated group (**c**). Mild cell infiltration and average airway epithelium and smooth muscle were observed in the 1 mg/kg Ech-treated mice (**d**).

The lung sections of control mice showed normal lung morphology. At the same time, the OVA challenge resulted in the marked recruitment of inflammatory cells, thickening of the bronchial epithelium, and smooth muscle thickening. Treatment with Ech in OVA-challenged mice ameliorated the lung morphology in a dose-dependent way, as seen in Figure 14.

### 2.13. Effect of Ech on Liver Histopathology

**Figure 15:**
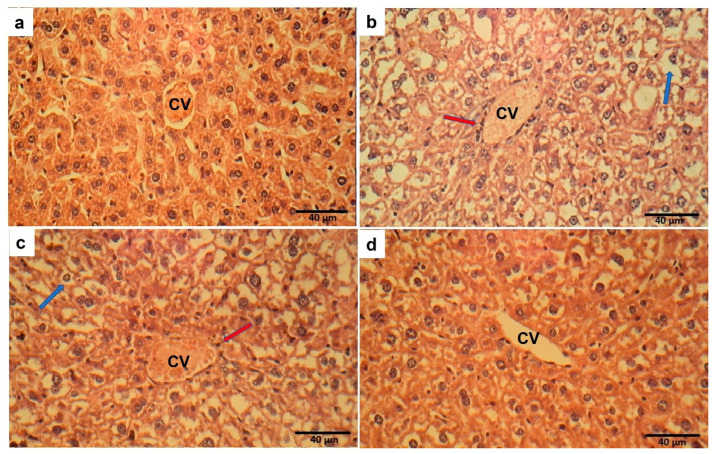
The control group (**a**) showed normal liver histology. Sections from a mouse in the OVA group (**b**) showed marked cytoplasmic vacuolation (blue arrow) with inflammatory cell infiltrations around the central vein (CV) (red arrow). Mild immune cell infiltration and vacuolation were noticed in the 0.1 mg/kg Ech-treated group (**c**). Normal liver tissue structure was observed in the 1 mg/kg Ech-treated mice (**d**).

The normal liver architecture of control mice was observed. In contrast, the OVA challenge resulted in marked cytoplasmic vacuolation with inflammatory cell infiltrations. Treatment with Ech in OVA-challenged mice attenuated these changes in tissue morphology in a dose-dependent way, as demonstrated in Figure 15.

### 2.14. Effect of Ech on Kidney Histopathology

**Figure 16:**
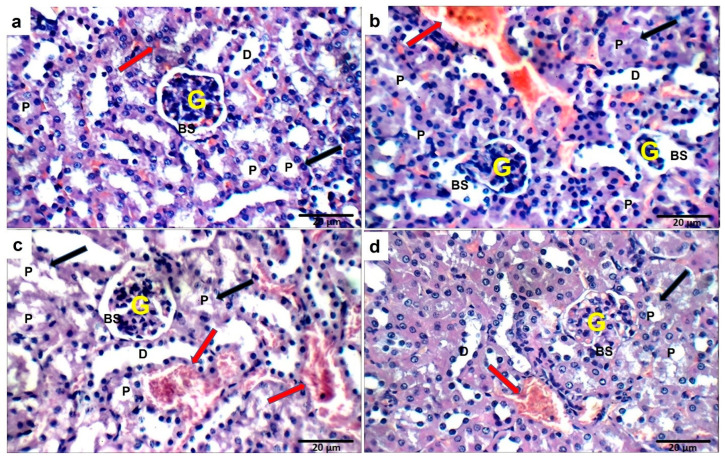
Kidneys from mice in the control group (**a**) showed normal-sized glomeruli (G) with average Bowman’s spaces (BS), proximal tubules (P) with average epithelial lining and preserved brush borders (black arrow), average distal tubules (D), and average interstitial blood vessels (red arrow). Sections from a mouse in the OVA group showed small-sized glomeruli with widened Bowman’s spaces, proximal tubules with scattered apoptotic epithelial lining and preserved brush borders, average distal tubules, and mildly congested interstitial blood vessels (**b**). No changes were observed after 0.1 mg/kg Ech treatment (**c**). Ameliorated kidney structure was observed in the 1 mg/kg Ech-treated mice (**d**).

The sections of control mice showed normal kidney structure. However, the OVA challenge resulted in structural abnormalities in the kidney tissue. Treatment with Ech (0.1 mg/kg) did not improve these alterations. However, treatment with 1 mg/kg Ech restored the normal kidney architecture, as shown in Figure 16.

### 2.15. Effect of Ech on Spleen Histopathology

**Figure 17:**
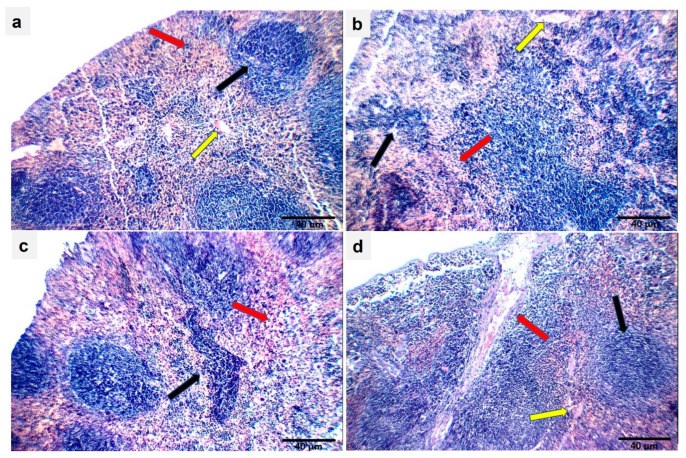
Sections of the spleens of mice in the control group (**a**) showed normal lymphoid follicles (white bulb) (black arrow), normal blood sinusoids (red bulb) (red arrow), and normal blood vessels (yellow arrow). Sections from a mouse in the OVA group showed small-sized lymphoid follicles (white bulb) (black arrow), expanded blood sinusoids (red bulb) (red arrow), and average blood vessels (yellow arrow). (**b**) Small-sized lymphoid follicles (black arrow) and markedly expanded congested blood sinusoids with many giant cells (red arrow) were observed after treatment with 0.1 mg/kg Ech. (**c**) Normal lymphoid follicles (white bulb) (black arrow), average blood sinusoids (red bulb) (red arrow), and mildly congested blood vessels (yellow arrow) were observed after treatment with 1 mg/kg Ech (**d**).

The sections of control mice showed normal spleen structure. On the other hand, the OVA challenge resulted in structural changes in the spleen’s morphology. Treatment with Ech in OVA-challenged mice reduced these changes in a dose-dependent way, as described in Figure 17.

## 3. Discussion

Allergic asthma is a chronic respiratory condition that affects many people and is caused by exposure to allergens. Lung inflammation increases the level of immune cell infiltration, increases the production of inflammatory mediators, and causes structural and functional alterations in the airways [19]. Currently, corticosteroids are among the primary medications used to manage asthma. However, these drugs can have notable negative consequences upon prolonged consumption [20]. Therefore, discovering a reliable asthma treatment is essential. The in vitro DPPH assay results demonstrated that Ech has antioxidant activity linked to its radical scavenging action. The heat-induced hemolysis assays also supported the anti-inflammatory properties of Ech. The in vitro findings showed that Ech possesses both antioxidant and anti-inflammatory activities.

Multiple irregularities in biological processes contribute to lung weight gain during persistent airway irritation. Numerous investigations have demonstrated that lung expansion results from increased smooth muscle in the lung during airway remodeling [21]. The lung weight can be utilized as a sign of tissue edema brought on by asthmatic inflammation and overproduction of mucus [22]. This study demonstrated a significant increase in the weights of the kidney, liver, spleen, and lung of OVA animals, which may be related to these organs’ oxidative stress and inflammation. On the other hand, administration of the Ech treatment restored the organs’ weight.

Typical clinical signs of asthma include wheezing, sneezing, and shortness of breath. Although it might be challenging to spot these symptoms in rodents, they exhibit symptoms like nasal scratching and fast breathing. The present study found that the comprehensive score of nasal scratching significantly increased in the untreated asthmatic group. However, following Ech treatment (1 mg/kg), the nasal scratching scores significantly decreased, showing the ameliorative effect of Ech on asthma symptoms.

Keap1 inhibitors impair the covalent link between Keap1 and Nrf2 to release Nrf2 transcriptional machinery that regulates its cellular antioxidant, cytoprotective, and detoxifying functions, protecting cells from oxidative-stress-mediated diseases [23]. Thus, Nrf2 activation offers cytoprotection against various pathologies, such as chronic lung and liver illnesses, autoimmune, neurological, and metabolic disorders, and cancer [24]. According to the outcomes of our molecular docking, Ech could stabilize Keap1. Our findings imply that Ech’s binding to Keap1 and disruption of the protein–protein interaction between Keap1 and Nrf2 may cause the nuclear translocation of Nrf2, which upregulates the expression of antioxidant molecules. Keap1 is an actin-binding protein with a molecular weight of 69.7 kD and 625 amino acid residues, 27 of which are cysteine residues [25]. Electrophilic drugs were initially developed to induce nuclear accumulation of Nrf2 by targeting its natural repressor protein Keap1 via covalent modifications on cysteine residues [26]. Quinones are a well-studied class of Nrf2 inducers [26]. Echinochrome A (7-ethyl-2,3,5,6,8-pentahydroxy-1,4-naphthoquinone) is a natural quinone that is oxidized to electrophilic quinones with a Michael acceptor group, permitting the thiol of Keap1 reactivity to bind to it [27,28]. Using RT-PCR and Western blot techniques, the gene and protein expressions of Keap1 and Nrf2 confirm molecular docking results.

The pathogenesis of asthma is mainly due to a disparity in the polarization of CD4+ Th cells. Increased Th2 cell activation is believed to play a pivotal role in asthmatic immunological responses, triggering and expanding inflammation through the secretion of a variety of Th2 mediators, including IL-4, IL-5, and IL-13 [29]. In our research, IgE, IL-4, and IL-1β serum levels increased significantly in the OVA-challenged mice. It has been reported that asthma is associated with a rise in IgE, IL-4, and IL-1β levels [30]. The increased level of these cytokines causes IgE synthesis and the infiltration of inflammatory immune cells, particularly eosinophils, that induce the release of ROS [31]. However, Ech’s treatment notably reduced their levels in a dose-dependent way.

Although asthma is a well-known lung condition, it also has a negative impact on other organs. More recent research has revealed the epidemiological association between asthma and a metabolic disorder [32]. Serum transaminases are sensitive biomarkers of liver cell destruction [33]. According to the study’s findings, the OVA group had significantly higher serum levels of AST and ALT and lower levels of total proteins and albumin than the control group. These results are consistent with previous investigations where OVA challenge led to higher AST and ALT levels and decreased levels of albumin and total proteins [34]. This rise in AST and ALT levels has been used as a sign of the severity of asthma, and it has been linked to the inadequate exchange of gases, which leads to hepatic oxygen deficiency and hepatocyte destruction [35]. Treatment with 1 mg/kg Ech has a significant protective effect against liver injury, as demonstrated by the lowering of serum AST and ALT levels and the elevation of albumin and total protein levels.

Asthma affects many chronic disorders, including chronic renal disease [36]. In the current study, kidney damage was shown by a significant rise in creatinine, urea, and uric acid in the mice that were given OVA compared to the control group. These findings align with previous research where the OVA challenge resulted in greater creatinine, uric acid, and urea concentrations [34]. It is possible to explain this increase in creatinine, urea, and uric acid due to the inflammatory response that can result in renal injury and dysfunction [37]. The current study further shows that treatment with 1 mg/kg Ech has a robust protective impact against kidney injury by decreasing the serum levels of creatinine, uric acid, and urea.

Oxidative stress plays a key role in the pathogenesis of multiple respiratory conditions, such as asthma [38,39]. It results from the disparity between the formation of free radicals and the antioxidant system. Elevated concentrations of free radicals in the lungs can cause functional and structural alterations and initiate biochemical cascades that are important in the pathogenesis of asthma [40]. Additionally, it promotes inflammation by increasing the secretion of proinflammatory cytokines and lowering antioxidant activity [41]. This study confirmed oxidative stress in different organs by increasing MDA and NO while GSH, SOD, GST, and CAT levels decreased. Collectively, it is possible to explain this increase in MDA and NO and the decline in GSH, SOD, GST, and CAT after OVA exposure as a result of disruption of the redox system and impaired antioxidant defenses, leading to the production of highly reactive free radicals, peroxidation of lipids, and cell destruction [42]. However, Ech treatment restored the balance between the oxidant and antioxidant defense mechanisms. Our study’s findings agree with a previous study, where the treatment with Ech reduced the level of oxidative stress parameters (MDA and NO) while increasing the concentration of GSH and the activity of CAT, SOD, and GST [43]. Furthermore, a recent study revealed that Ech-A inhibits sulfide catabolism and H2S/HS^-^ formation in hypoxic and inflammatory cells [44]. Thiyl radicals, disulfides, sulfenic acids, and disulfide oxides are reactive sulfur species that rapidly oxidize and inhibit thiolproteins and enzymes [45].

## 4. Materials and Methods

### 4.1. Reagents

Standard Echinochrome (Ech) (Vladivostok, Russia) and Dulbecco’s phosphate buffer saline (PBS) 10× (SEROX GmbH^®^, Mannheim, Germany) were used. Ovalbumin (OVA), aluminum hydroxide, dimethyl sulfoxide (DMSO), tris ethylenediaminetetraacetic acid (EDTA), bovine serum albumin (BSA), hydrochloric acid (HCl), diethyl ether, and anhydrous sodium sulfate were obtained from Sigma-Aldrich (St. Louis, MO, USA). Biochemical kits were obtained from Bio-diagnostics Company (Giza, Egypt). Total IgE (Cat No. E-20550Mo) (Houston, TX, USA), IL-4 (Cat No. BMS613), and IL-1β (Cat No. BMS6002) (Invitrogen by Thermo Fisher Scientific, Waltham, MA, USA) were obtained. TaqMan probes and TaqMan Gene Expression Mastermix (Thermo Fisher Scientific) were obtained. Antibodies against Nrf2, Keap1, and HRP-conjugated goat anti-rabbit secondary antibody were obtained from (Abclonal Technology, Wuhan, China). Antibody against GAPDH was obtained from Cell Signaling Technology (Danvers, MA, USA).

### 4.2. Ech Extraction

Sea urchins (*Paracentrotus lividus*) were gathered from the Mediterranean shore of Alexandria (Egypt) and transferred to the lab on ice. The samples were properly cleaned with seawater to remove sand and overgrown organisms at the collection location and transferred to the lab. Taxonomic guides recognized the specimens [46]. The specimens were shade-dried immediately. Ech was extracted according to the method adopted by Amarowicz et al. [47] with modifications. Spines were removed, and then the shells were opened into 2 pieces using scissors to remove the animal’s internal structures under constant tap water flow. The spines and shells were allowed to air-dry for three days in a cold, dark place. Dried samples were ground into powder. After this, the obtained powder was slowly added to a certain amount of 6 M HCl. Then, the obtained solution was filtrated before extracting the echinochrome pigment several times with diethyl ether. Then, a suitable amount of sodium sulfate (anhydrous) was added to remove the water before evaporating the ether using a rotatory evaporator. Ech was eventually obtained and kept at −20 °C. Detailed information about FTIR and mass spectroscopy, UV, ^1^H NMR, and ^13^C NMR spectra of Ech is described in the Supplementary File.

### 4.3. HPLC Analysis

A Shimadzu HPLC system (Kyoto, Japan) was used, which included two LC20AD pumps, a DGU-20 A3 degasser, and an SPD-M20 A diode-array detector. With a 1.0 mL/min flow rate, a Zorbax Eclipse Plus C18 column (250 mm 4.6 mm, 5 m) was employed for chromatographic separation with acetonitrile/methanol (5:9 *v*/*v*) and 0.1% formic acid as the binary mobile phase. An elution profile was as follows: formic acid containing 40–70% acetonitrile for 0–25 min (linear gradient). The volume of the injection was 20 μL. Between 200 and 800 nm, the detection was noted. The LC Solution (Shimadzu) was the data analysis system. DMSO was used to dissolve Ech at a of 5 mg/mL concentration.

### 4.4. In Vitro Biological Studies

#### 4.4.1. Antioxidant Activity Using DPPH Radical Scavenging Protocol

Ech (20, 40, 60, and 100 μg/mL) in ethanol was combined with 1500 μL of 0.1 mM DPPH–ethanol solution. After 30 min of incubation at ambient temperature, the DPPH free radical was reduced by reading the absorbance at 517 nm [48,49]. Positive control included ascorbic acid. The following equation calculated the inhibition ratio (percent):

% RSA = (absorbance of control − absorbance of sample)/(absorbance of control) × 100.

#### 4.4.2. Anti-Inflammatory Activity Using Heat-Induced Hemolysis Protocol

Venipuncture blood from healthy volunteers was centrifuged at 3000 rpm for 10 min at 4 °C in heparinized tubes. RBCs were washed three times in PBS after plasma removal. RBCs were resuspended in 10 mL PBS. The assay combination included 0.5 mL of RBC suspension and 0.5 mL of Ech (100, 200, 300, 50 µg). A UV–visible spectrophotometer at 560 nm evaluated supernatant hemoglobin [50]. Diclofenac was used as the control. This equation estimates hemolysis percentage:

Protection % = 100 − [(Optical density of sample)/(Optical density of control) × 100]

### 4.5. Molecular Docking Interaction between Ech and Keap1

The crystal structure of the Kelch-like ECH-associated protein 1 (Keap1) was obtained from the Protein Data Bank with PDB ID: 4L7B. Using the molecular visualization software, Pymol [51], solvent molecules, heteroatoms, and other experimental inhibitors were removed from the target macromolecule. Hydrogen bonds were added, and the file was extracted in PDB format. The grid box dimensions of the binding site were then determined with Keap1’s cocrystallized ligand as its center [52]. The structures of the target protein file and the cocrystallized ligand were converted into the PDBQT format using Auto-Dock (MGL Tools) [53]. Ech structure was downloaded in sdf format from PubChem and converted into the PDBQT using Open Babel software [54]. Virtual docking was performed using Auto-Dock Vina [55]. The results were exported as comma-separated files (CSV). The mean lowest binding energy was used to predict the binding affinity of echinochrome with Keap1. Pymol and the Protein–Ligand Interaction Profiler were used to visualize three-dimensional hydrophobic interactions [56]. Two-dimensional interactions were viewed with Discovery Studio (BIOVIA, San Diego, CA, USA).

### 4.6. Experimental Animals

The National Research Center (Giza, Egypt) provided 32 female BALB/c mice (Mus musculus) weighing (18–22 g). They were fed, kept in groups in sterile enclosures, and had free access to water *ad libitum*. The Institutional Animal Care and Use Committee (IACUC) at Cairo University in Egypt approved this research with the number CU/I/F/32/22. The instructions for the care and use of laboratory animals were used in all of the experiments in accordance with international guidelines.

### 4.7. Animal Grouping and Experiment Design

The experimental procedure used in this research to induce asthma was established according to Bai et al. [57], with some modifications. For sensitization, the mice were intraperitoneally injected with 20 μg of OVA mixed with 1 mg aluminum hydroxide gel in sterile PBS (pH 7.4) (200 μL final volume). For the challenge, the mice were exposed once daily to inhalations with 2.5% OVA on days 21, 22, and 23. After two weeks of acclimatization, mice were randomly assigned to 4 groups—a control group, an OVA group, a low-dose group of Ech (0.1 mg/kg) [58], and a high-dose group of Ech (1 mg/kg)—each of which contained 8 mice. One hour before the challenge, Ech (0.1 and 1 mg/kg) was intraperitoneally administered. Mice received the same volume of PBS as the control group. Mice were sacrificed after the last challenge.

### 4.8. Evaluation of Body Weight and Nasal Scratching

Body weight gain and the weight of the lung, spleen, kidney, and liver of all mice were recorded at the end of the experiment. Nasal scratching was assessed and scored for 10 min following the final challenge, with 2.5% OVA on the final day. The results were as follows: Mice who scratched their noses 0–2 times received a score of 0, 3–5 times received a score of 1, 6–8 times received a score of 2, and 9 or more times received a score of 3 [59] (an illustration video is attached as Supporting Material).

### 4.9. Sample Collection

Twenty-four hours after the last OVA challenge, mice were isoflurane-anesthetized and blood was taken from the retro-orbital plexus to assess the liver (ALT, AST, albumin, and total protein) and kidney (creatinine, urea, and uric acid) function biomarkers and measure the serum concentrations of IgE, IL-4, and IL-1β. The spleen, kidney, and liver were removed, weighed, and homogenized for evaluating Nrf2, Keap1, and oxidative stress biomarkers. At the same time, the lung, liver, kidney, and spleen were utilized for evaluating OVA-induced histopathological alterations using H&E.

### 4.10. Measurement of Serum Levels of IgE, IL-4, and IL-1β

IgE, IL-4, and interleukin 1β (IL-1β) were quantified according to the instructions of the kits and expressed as pg/mL using an ELISA plate reader (DAS Instruments, model A3, Rome, Italy).

### 4.11. Evaluation of Serum Biochemical Parameters

The serum levels of ALT, AST, total protein, albumin, creatinine, uric acid, and urea were evaluated according to the kit’s instructions.

### 4.12. Western Blot Assays

The tissue homogenates were mixed with protein lysis buffer (50 mM Tris-HCl pH 7.5, 150 mM NaCl, 5 mM EDTA, 0.5% Triton X-100, and protease inhibitor). Samples were sonicated and centrifuged for ten minutes at a speed of 10,000 rpm at 4 °C. The samples were then subjected to 10% SDS-PAGE gel electrophoresis (50 µg of protein per lane), and the Bradford technique assay was used to determine the protein content. After transferring proteins to PVDF membranes, they were blocked for 1 h at room temperature with blocking buffer (TBST buffer with 5% skim milk powder), followed by the addition of the primary antibodies Keap1, Nrf-2 (1:1000), and GAPDH (1:2000), which were then incubated overnight at 4 °C. After washing with TBST, secondary antibodies (1:5000) were applied to the membranes. They were incubated for 1 h at room temperature. Enhanced chemiluminescence (ECL; Thermo Scientific; Shanghai, China) was used to observe the blots. All reactions were performed in triplicate and the blots were quantified and assessed using ImageJ (version 1.8.0, Bethesda, MD, USA).

### 4.13. Real-Time Reverse Transcriptase-Polymerase Chain Reaction (RT-PCR)

RT-PCR was used to analyze the amount of mRNA expression for both Nrf2 and Keap1. The liver, kidney, and spleen homogenates were treated with the triazole reagent and subjected to the standard protocol for isolating total RNA. A Sensi-Script Reverse Transcriptase Kit (Qiagen Hilden, Germany) was used to convert RNA to cDNA. With the use of TaqMan probes, we were able to conduct quantitative PCR. TaqMan Gene Expression Master Mix was used for qPCR using TaqMan primers for mouse Nrf2 and Keap1. The CFX384 Touch Real-Time PCR Detection System (BioRad, Mississauga, ON, Canada) was used for the quantitative RT-PCR. Using the delta–delta Ct technique, we calculated the abundance of each transcript. All reactions were performed in triplicate and the mean value was used to calculate expression levels after normalization against β-actin.

### 4.14. Determination of Liver, Kidney, and Spleen Oxidative Stress Biomarkers

The kidney, spleen, and liver tissues were homogenized in an ice-cold 0.1 M Tris HCl buffer, pH 7.4, and centrifuged for 15 min at 3000 rpm [60,61]. The supernatant was utilized for evaluating the concentration of GSH and the activities of CAT, SOD, and GST enzymes in the liver, kidney, and spleen tissues. Additionally, the MDA and NO were measured according to the instructions of commercial kits.

### 4.15. Histopathological Examination of Lung, Kidney, Liver, and Spleen

Mice were dissected, and the lung, kidney, spleen, and liver were removed entirely and fixed in 10% formalin for 24 h. Sample sections were cut, stained with hematoxylin and eosin (H&E), and examined under a light microscope [62].

### 4.16. Statistical Analysis

For the statistical analyses, IBM’s Statistical Package for the Social Sciences (SPSS) was utilized. Mean and SEM were used to express values. A one-way analysis of variance (ANOVA) was used to determine group differences. The graphs were made utilizing the 8th version of GraphPad Prism. The group means were compared using Duncan’s post hoc test, and *p* < 0.05 was regarded as statistically significant.

## 5. Conclusions

We found that Ech reduced the severity of asthma-induced immunological and physiological alterations in mice. Treatment with Ech also improves the histology of the spleen, lungs, kidneys, and liver of asthmatic mice. Inhibition of inflammation and oxidative stress are two of Ech’s therapeutic actions in managing asthma by modulating the Keap1/Nrf2 signaling pathway.

## Figures and Tables

**Table 1:** The docking interaction data calculations of Ech and Keap1.

Protein–Ligand Interactions Profile					
Index	Residue	Amino Acid	Distance (Å)	Ligand Atom	Type of Interaction
1	334B	TYR	3.54 (3.08)	19 O [O3]	Hydrogen bond acceptor
2	363B	SER	2.95 (2.06)	16 H [O2]	Hydrogen bond acceptor
3	382B	ASN	3.88 (3.36)	21 O [O3]	Hydrogen bond acceptor
4	387A	ASN	3.08 (2.15)	19 O [O3]	Hydrogen bond donor
5	414B	ASN	4.04 (3.24)	16 H [O2]	Hydrogen bond donor
6	602B	SER	2.88 (2.35)	17 O [O3]	Hydrogen bond acceptor
7	334B	TYR	3.85	1 O	Hydrophobic interaction
8	572B	TYR	3.79	2 O	Hydrophobic interaction
9	577B	PHE	3.63	1 O	Hydrophobic interaction
10	334 B	TYR	3.88	5 C, 6 C, 7 C, 8 C, 9 C, 10 C	π-Stacking

## Data Availability

Not applicable.

## Supplementary Materials

The following supporting information can be downloaded at: https://www.mdpi.com/article/10.3390/md21080455/s1, Video S1: Nasal Scratching.
